# A Comparison of Laser and Mechanical Surface Pretreatment Methods on Shear Bond Strength of Resin Composite to Resin-Modified Glass Ionomer

**DOI:** 10.30476/dentjods.2022.92281.1624

**Published:** 2023-03

**Authors:** Faramarz Zakavi, Sarah Gholizadeh, Sana Dibazar, Mehdi Esmaeili

**Affiliations:** 1 Dept. of Operative and Esthetic Dentistry, Dental Faculty, Ahvaz Jundishapur Medical Science University, Ahvaz, Iran; 2 Dept. of Operative and Esthetic Dentistry, Dental Faculty, Tabriz University of Medical Science, Tabriz, Iran; 3 Dept. of Orthodontics Dentistry, Dental Faculty, Tabriz Azad University of Medical Science, Tabriz, Iran

**Keywords:** Lasers, Glass ionomer cements, Composite resins, Bond strength

## Abstract

**Statement of the Problem:**

Dentin margins have a higher microleakage than enamel in composite restorations due to weaker bond durability. Clinically, most margins are located apical to cementoenamel junction on the dentin or cementum. Different surface preparation methods may have dissimilar effects on the shear bond strength of resin-modified glass ionomer (RMGI) to composite resin, regarding the layering technique employed in restoration of these cavities.

**Purpose:**

This study aimed to investigate the shear bond strength of RMGI to composite resin using different mechanical surface preparation methods.

**Materials and Method:**

In this *in vitro* study, 72 RMGI samples were prepared in six groups (n=12): control, acid etching, air abrasion with aluminum oxide (Al_2_O_3_), bur, Er: YAG laser, and Er, Cr: YSGG laser groups. Two samples from each group were randomly examined under a scanning electron microscope (SEM). A bonding layer and composite resin were applied and cured. Then the samples were subjected to 5000-cycles thermocycling procedure. The shear bond strength was then evaluated using a universal testing machine. Finally, the failed surface of the samples was evaluated under a light microscope to examine the failure mode. For the statistical analysis, one-way ANOVA and Games-Howell post hoc test was used to compare quantitative variables and chi-squared test to investigate the relationship between the failure mode and the groups. The significant level was set at 0.05.

**Results:**

The results showed significant differences between the mean of the strength variable in the groups (*p*< .001). The acid-etch and air abrasion groups exhibited significantly the lowest and highest values, respectively. The chi-squared test results also showed a significant relationship between the failure mode in the study groups (*p*= .008).

**Conclusion:**

Acid etching before completing RMGI polymerization could adversely affect shear bond strength. The air abrasion and bur methods are among the best methods to increase the shear bond strength between composite resin and RMGI. Using Er, Cr: YSGG and Er: YAG lasers could increase the bond strength results.

## Introduction

Patients' increasing demands for esthetic procedures and recent advances in adhesives have made composite resins an essential component of modern dentistry. Therefore, composite resins have recently been introduced as the first choice for direct restorative procedures in anterior and posterior teeth [ 1 - 2 ]. In addition to their many advantages, the polymerization shrinkage problem can disrupt marginal sealing in areas with weak bond durability [ 3 ]. Most of the restoration margins in the clinic are apical to the cementoenamel junction on the dentin or cementum. This weak durability of the bond to dentin and cementum at the margins of these restorations and other factors, such as the difficulty in moisture control and insufficient access to deep cavities, facilitate sealing failure and endanger the long-term efficacy of restorations [ 4 ]. The layering technique on a layer of glass-ionomer cement has been suggested to reduce polymerization shrinkage, microleakage, and clinical longevity of restorations [ 5 - 6 ].

Caries and an improper bond between glass-ionomer and composite resin are the main factors for the failure of such restorations [ 7 - 8 ]. Recently, conventional glass-ionomer cement has been replaced mainly by resin-modified glass ionomer (RMGI) cement because of some advantages, including higher physical and chemical properties [ 9 ]. RMGI cement contains pendant methacrylate groups and 2-hydroxyethyl methacrylate (HEMA) [ 10 ]. The similarity in chemistry and curing mechanisms by the free radical initiator system results in a chemical bond to composite resin [ 11 - 12 ]. The HEMA molecules and the un-reacted methacrylate groups could also lead to superior chemical covalent bonds [ 13 ]. HEMA is a hydrophilic molecule that increases the surface wettability [ 14 ] and makes the RMGI material more likely to absorb water [ 15 ]. This affinity could make the RMGI surface more susceptible to contamination during the restoration of deep margins [ 16 ]. Water absorption also may lead to dilution of monomers in uncured material to the extent that it interferes with the polymerization [ 17 ] and compromises the chemical bond mechanism. Besides, contamination of bonding surfaces with oral fluids could profoundly affect bond strength results [ 18 ]. In some cases, even using conventional etching methods could not restore the original values [ 19 ]. 

On the other hand, despite the studies supporting the efficiency of chemical bonding for the durability of restorations [ 11 - 12 ], due to different thermal expansion coefficients of RMGI and composite resin, some reduction in the bond strength values is possible [ 20 ]. Studies have indicated a decrease in bond strength of RMGI to composite resin and adhesive failures after thermocycling [ 15 , 21 ]. Considering dynamic conditions of the oral cavity, simultaneous use of micromechanical methods [ 9 , 20 ] and chemical bonds in deep margins, could enhance the bond values to a proper level even when the clinician was not aware of the contamination. There are some methods to enhance micromechanical bonds of the RMGI surface. Acid etching as the conventional method [ 12 ] and aluminum oxide (Al_2_O_3_) particles in the air abrasion method improve surface energy and the area for bonding, resulting in enhanced bond strength [ 22 ]. Lasers have been introduced to increase surface roughness due to the contradictory results of previous studies on the use of acid etching on RMGI bond strength, time loss, limitations on the expiration date of acid etchants, and the sensitivity of their technique [ 23 - 24 ].

Er: YAG and Er, Cr: YSGG lasers are commonly used for etching in dentistry [ 24 ]. These lasers have a moderate energy level, which prevents melting or changes in the crystal structure of materials [ 25 ].

Numerous studies have shown the favorable effect of these methods on bond strength, following an increase in surface energy and the effective interfacial surfaces between different substrates [ 26 - 28 ]. However, limited studies have been performed on the effect of these methods on RMGI [ 22 - 23 , 29 ]. This study investigated and compared the effect of different mechanical roughening methods on the shear bond strength of RMGI to composite resin.

## Materials and Method

In this experimental study, 72 cylindrical samples of RMGI (Fuji II LC; GC Corporation, Tokyo, Japan) with A2 shade were prepared using plastic molds. Table 1 shows the characteristics of the materials used. After mixing the powder and liquid according to the manufacturer’s instructions, a layer of mixed material was placed inside a plastic mold measuring 3mm in diameter and 5 mm in height. Each sample was covered with a celluloid strip (Maquira Dental Products, Maringa, Brazil), placed on a translucent glass slab, and cured for 20 seconds with a light-curing unit (Astralis 7; Ivoclar Vivadent, Amherst, NY, USA) at 400mW/cm^2^ intensity from both sides. The device's tip was perpendicular to the surface of the samples. The intensity of the curing device was measured periodically after curing every 10 samples, using a radiometer device (Demetron/Kerr Corp, Orange, CA, USA). The plastic mold was cut using a surgical blade (Medico International Trading Co. LTD, China). The extracted samples were randomly divided into six groups (n=12):

Group 1: No surface preparation (control group) was performed.

Group 2: Acid etching with 37% phosphoric acid (Morva Etch, Morvabon, Iran) was done for 15 seconds followed by rinsing the acid for 10 seconds with distilled water and removing the excess water with a wet cotton pellet.

Group 3: The surfaces of the samples were roughened with 30-μm Al_2_O_3_ particles (Microblaster Dento-Prep™, Dental Microblaster, Denmark) for 10 seconds; then, the samples were irrigated for 10 seconds with distilled water and the excess water was removed with a piece of wet cotton pellet.

Group 4: The surfaces of the samples were roughened with a rough diamond bur (012 Cylinder Flat End, SS White Burs, Inc. Lakewood, NJ) for 3 seconds at high-speed under water spray. Then, the samples were irrigated for 10 seconds with distilled water, and excess water was removed with a wet cotton pellet.

Group 5: The surfaces of the samples were roughened using Er: YAG laser (M021-3AF/4, Fotona, Slovenia) with 1064-nm wavelength, 1.5-W power, 5-Hz frequency, 8% water output, and 4% air output from a 10-mm distance and with 300-mJ energy in the micro-short pulse mode. Then the samples were irrigated for 10 seconds with distilled water and the excess water was removed with a wet cotton pellet.

Group 6: The surfaces of the samples were roughened using Er, Cr: YSGG laser (Water Lase iPlus, Biolase, USA) with MZ8 tip measuring 800 µm in diameter, 0.502-mm^2^ spot-size, 2780-nm wavelength, 1-W power, 20-Hz frequency, 20% water output, and 10% air output at 1-mm distance from the surface for 15 seconds with 53.07J/cm^2^ intensity. The samples were then irrigated for 10 seconds with distilled water and the excess water was removed with a wet cotton pellet.

**Table 1:** The characteristics of the materials used

Manufactured by	Lot No.	Description and composition	Material/ Type
GC corporation, Tokyo, Japan	1801061	Fluoro-alumino-silicate glass, Polyacrylic acid, HEMA, trimethlyene dicarbonate, and other proprietary ingredients	RMGI Fuji II LC
GC corporation, Tokyo, Japan	190307B	UDMA, SiO2, fluoro-alumina-silicate glass, pre-polymerized filler	Gradia direct
3M ESPE, St. Paul, MN, USA	N869156	BISGMA, HEMA, copolymer of acrylic and itaconic acids, water, ethyl alcohol, glycerol 1, 3-dimethacrylate, diurethane dimethacrylate, silane treated silica, water	Adper Single Bond 2
Morvabon, Iran	101007169	37% Phosphoric acid	Morva acid etch

Two samples from each group were randomly examined under a scanning electron microscope (SEM) (FESEM; HITACHIS-4160, Japan) to evaluate the surface microscopically. Then a bonding layer (Adper Single Bond 2; 3M ESPE, St. Paul, MN, USA) was applied on the surfaces of all the samples according to the manufacturer’s instructions and cured for 10 seconds with 400 mW/cm^2^ intensity. 

Composite resin (Gradia Direct Anterior, GC Co., Tokyo, Japan) with A1 shade was applied in one layer of 2mm and cured for 40 seconds beyond the translucent glass slab and perpendicular to the surface. Celluloid tapes were used to prevent the adhesion of the composite resin to the glass slabs and achieve a smooth surface. The light intensity was measured periodically for every 10 samples using a radiometer device.

To simulate clinical condition and absorbing water, samples were subjected to distilled water at 37°C for 24 hours, and then they underwent a 5000-cycles thermocycling procedure (TC/300; Vafaei Industrial, Tehran, Iran) to induce aging process.

The shear bond strength was evaluated using a universal testing machine (Santam, STM-20, Iran) by placing a chisel at the resin-RMGI interface at 1mm/min crosshead speed. Finally, the fractured surfaces of the samples were studied under a light microscope (Motic SMZ -143 SERIES, Micro-optic industrial group Co, Xiamen, China) to evaluate the fracture mode (cohesive, adhesive, and mixed) using the color difference between the composite resin and RMGI. One-way ANOVA (SPSS 20) was used to compare the quantitative variables (bond strength). The significant level was set at 0.05.

## Results

Table 2 shows the means and standard deviations of the strength variable of the groups.

**Table 2:** Means and standard deviations of the strength variable of the study groups

Group	N	Mean	Std. Deviation	Std. Error	95% Confidence Interval for Mean		Min.	Max.	*p*
					Lower Bound	Upper Bound			
Control	10	16.8000	4.65666	1.47256	13.4688	20.1312	10.80	22.70	<.001
Acid etch	10	15.2000	3.61017	1.14164	12.6174	17.7826	11.20	19.80	
Al_2_O_3_	10	28.4000	8.10322	2.56246	22.6033	34.1967	19.40	37.20	
Bur	10	25.1000	5.56936	1.76119	21.1159	29.0841	18.30	31.70	
Er YAG	10	18.0200	3.86603	1.22255	15.2544	20.7856	13.40	23.70	
Er-Cr YSGG	10	17.2200	3.36016	1.06258	14.8163	19.6237	12.60	21.80	

**Figure 1:**
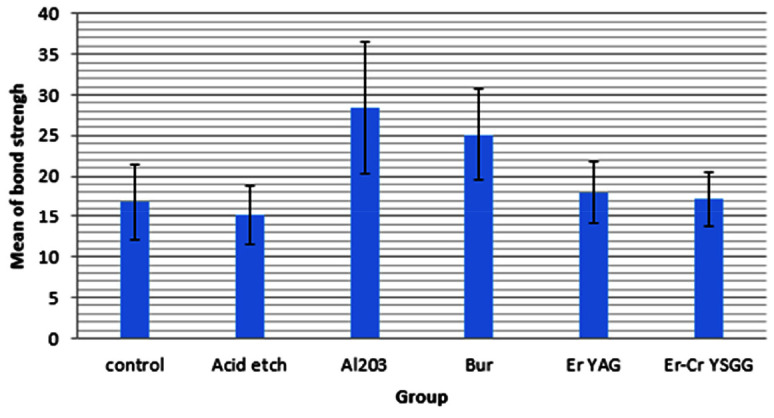
Bar chart of the mean strength in the groups

A parametric test was used to analyze the null hypothesis because of the normal distribution of the bond strength. Leven test was used to evaluate the homogeneity of the variance in the groups. The significant level was set at *p*< 0.05. This test showed that the equality of variances in the groups was rejected, and there were significant differences between the variances of the groups (Figure 1).

One-way ANOVA was used to evaluate the difference between the mean of the strength variable in the groups. The significant level of the test was set at *p*< 0.05. The results showed a significant difference between the mean of the strength variable in the groups (*p*< .001).

The Games-Howell post hoc test was used to determine which of the two groups were different. The significant level of the test was set at *p*< 0.05. Table 3 shows the results.

**Table 3:** Games-Howell test results

(I) Group	(J) Group	Mean Difference (I-J)	*p*
Control	Acid etch	1.60000	.951
	Al_2_O_3_	-11.60000*	.015
	Bur	-8.30000*	.021
	Er YAG	-1.22000	.986
	Er-Cr YSGG	-.42000	1.000
Acid etch	Al_2_O_3_	-13.20000*	.005
	Bur	-9.90000*	.003
	Er YAG	-2.82000	.557
	Er-Cr YSGG	-2.02000	.784
Al_2_O_3_	Bur	3.30000	.889
	Er YAG	10.38000*	.028
	Er-Cr YSGG	11.18000*	.016
Bur	Er YAG	7.08000*	.043
	Er-Cr YSGG	7.88000*	.017
Er YAG	Er-Cr YSGG	.80000	.996

* The mean difference is significant at the 0.05 level

There was a significant difference between the mean strength variable in the bur and Al_2_O_3_ groups and the mean in control, acid etch, Er: YAG, and Er, Cr: YSGG groups and the mean strength variable of the bur and Al_2_O_3_ groups were significantly higher than that in other groups (Table 3).

**Figure 2:**
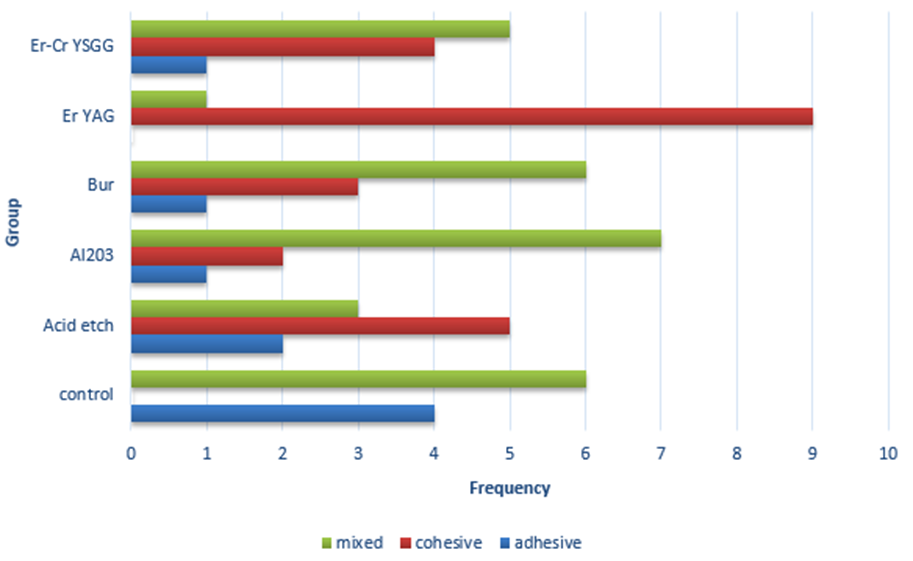
Bar chart of the frequency of the samples in terms of the failure mode in the groups

There was no significant difference between the mean strength variables in other groups. The prevalence of the samples according to the failure mode in the groups is shown in Table 4 and Figure 2.

**Table 4:** The frequency of the failure mode in the groups

Group	Adhesive	Cohesive	Mixed	*p*
Control	4	0	6	.008
Acid etch	2	5	3	
Al_2_O_3_	1	2	7	
Bur	1	3	6	
Er YAG	0	9	1	
Er-Cr YSGG	1	4	5	

A chi-squared test investigated the relationship between the failure mode and the groups. The significant level of the test was set at *p*< 0.05. The chi-squared test results showed a significant relationship between the failure mode and the groups (*p*= .008). The frequency of adhesive failure in the control group, the frequency of cohesive failure in the Er: YAG group and the frequency of mixed failure in the Al_2_O_3_ group were significantly higher than in other groups.

## Discussion

The present study results showed that different methods of mechanical surface preparation for RMGI significantly affect the final bond strength to composite resin. The lowest values were recorded in the acid etch group, and the highest values were recorded with the air abrasion with 30-µm Al_2_O_3_ particles. The mean shear bond strength values, in descending order, were recorded in the bur, Er, Cr: YSGG laser, Er: YAG laser, and the control groups. 

The results of etching the surface of the samples with 37% phosphoric acid were lower than the control group. However, this difference was not significant. The decrease in bond strength following the application of phosphoric acid was consistent with the results of previous studies, which showed that the total-etch system has lower shear bond strength than the self-etch system with weaker acidity [ 9 , 30 ]. According to the results of another study [ 5 ], no change in bond strength was observed between RMGI and the composite resin, which was attributed to the possibility of no acid penetration due to the presence of polymer in the RMGI structure.

**Figure 3:**
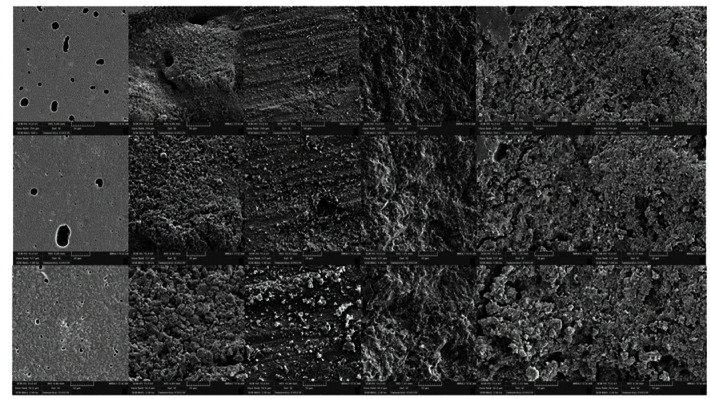
control, acid etch, bur, air abrasion, Er YAG, Er-Cr YSGG

Fuji II LC comprises glass-ionomer as the main composition with some resin components, including HEMA, bisphenol A-glycidyl methacrylate, and photoinitiators. HEMA plays an essential role in the RMGI photo-polymerization reaction [ 31 - 33 ]. If this monomer remains unpolymerized, it penetrates and increases the surface wettability by the resin bonding agent and composite resin, consequently increasing the formation of the resin tags [ 10 ]. The presence of un-reacted methacrylate groups that remain in the polyacid chain during polymerization makes it possible to form strong covalent bonds with the resin bonding agent [ 10 ]. Since it is not possible to wait for a long time in the presence of the patient, the application of early acid-etching would result in some ion release [ 34 ], especially in the surface areas of the glass, and reduce the overall strength of the glass lattice along with the elimination of un-reacted chains [ 13 , 35 ]. This explains the bubbles and cracks visible in SEM evaluations (Figure 3).

Evaluation of the failed surfaces showed that most cases had a cohesive failure in RMGI, and a small number of samples had adhesive failure mode. To justify mixed and adhesive failures, it may be suggested that HEMA and un-reacted methacrylate groups be removed from the RMGI surface in some areas. Cohesive failure in the glass matrix and low bond strength results are reasons for the weak substrate matrix after acid application and early irrigation. Due to the application of 37% phosphoric acid, the outer layer of the material is weakened, and its tensile strength is reduced [ 10 ].

The results also showed that in the samples prepared with a bur, the shear bond strength of RMGI to composite is significantly higher than the control, acid, Er: YAG and Er, Cr: YSGG groups.

Bur roughening will create mechanical porosities and increase the bond strength. A study on the shear bond strength between RMGI and nano-ionomer with composite resin showed that preparation with a carbide or diamond bur increased the bond strength [ 29 ]. Another study on the repair bond strength of cured laboratory composite resin with new composite showed that roughening with a diamond bur, although less than other methods increase the bond strength [ 26 ]. The use of a bur causes macroscopic and microscopic irregularities in the composite surface and effectively increases the bond strength between the two surfaces [ 36 ]. Microscopic irregularities are more effective in bond strength through penetration of resin bonding agents and the formation of micro-tags than macroscopic irregularities [ 37 - 39 ]. These findings were consistent with the results of a recent study in which, in the bur-prepared group, the bond strength increased significantly, and most fractured substrates showed mixed and cohesive failure. 

Due to less uncured methacrylate monomers, the RMGI surface is more abraded and roughened than the laboratory composite resin abraded by a bur [ 26 ]. It should be noted that bur abrasion creates a smear layer that is not present in the other groups.

According to the bond failure mode results, most failures were mixed. The presence of the smear layer reduces the chemical bond after eliminating the surface layer and is against the increasing surface roughness, which causes debonding in some surface areas. 

The highest bond strength was attributed to the samples air abraded with Al_2_O_3_ particles, and the differences from the laser, control, and acid etch groups were significant. A study on the shear bond strength of resin cement to indirect composite resin showed that air abrasion increases the bond strength in the samples [ 40 ]. Another study [ 27 ] on the repair bond strength of indirect composite resin to direct composite restorations showed that the air abrasion method with 50-µm Al_2_O_3_ particles, according to the results of the present study, significantly increases the bond strength.

A study [ 22 ] on the shear bond strength of RMGI to composite resin showed that air abrasion would increase the bond strength values less than acid etching and Er, Cr: YSGG laser methods. They also showed that the application of acid etching and a laser resulted in the highest bond strength values, respectively. Different RMGI surface preparation methods increase the surface roughness of the material surface, increasing the bonding surface area (Figure 3). 

In the present study, the samples were aged under thermocycling after preparation to simulate the clinical condition. By examining the results of a recent study and comparing it with another study [ 23 ], it was concluded that acid etching and lasers, apart from increasing the surface roughness, cause changes in the surface structure of RMGI, and these changes are more pronounced after aging.

According to SEM results, the RMGI surface from air abrasion has homogeneous micro-porosities throughout the surface (Figure 3), which allows the formation of resin micro-tags for the adhesive bonding agent and composite resin [ 37 - 39 ]. Similar to bur roughening, air abrasion abrades some parts of the surface. However, due to the small size of Al_2_O_3_ particles and the use of the minimum size in the present study, the surface changes are homogeneous and microscopic and the presence of the smear layer is minimal. Examining the bond failure mode showed that most samples underwent mixed failures, with few cohesive failures. These results and the high bond strength results indicate an adequate bond between the two substrates. 

The samples prepared with Er: YAG and Er, Cr: YSGG lasers exhibited higher bond strength than the control group. The bond strength values of Er: YAG group were higher than the Er, Cr: YSGG group. However, the differences were not significant compared to each other and the control group. The laser initiates its mechanism by evaporating water and other hydrated structures such as hydroxyl groups [ 41 ]. It should be noted that after the evaporation of these components, increased heat could damage the substrate. Therefore, areas with more hydrated components and, to some extent, some mineral components will be affected by the laser [ 42 ]. The polymerized RMGI structure contains an ion matrix resulting from an acid-base reaction and a polymerized matrix resulting from the free radical reaction. The penetration of the laser beam and its effect, especially on the ionic matrix, will cause micro-porosities on the surface, consequently increasing the bond strength compared to the control group. These results are consistent with the study of Navimipour *et al*. [ 23 ], who showed that applying Er, Cr: YSGG laser on the surface of RMGI samples will increase the bond strength.

According to previous studies, laser application creates a layer of small spots on the surface of the substrate. In the present study, these spots were also observed on the sample surfaces after laser application. Although, according to the recommendations of the previous study [ 23 ], before applying the bonding, the stains were removed with a wet cotton pellet. Examination of the surface of the samples under a light microscope showed that these strains were much more numerous in the Er: YAG laser group than the Er, Cr: YSGG group. Considering the higher shear bond strength in the Er: YAG group than the Er, Cr: YSGG group, it can be concluded that the Er: YAG laser has a higher ability to create porosity on the RMGI surface than the Er, Cr: YSGG laser. 

Despite the ability to create irregular microporosities in the RMGI matrix [ 23 ], laser can affect the chemical and ionic structure of RMGI. The heat generated by the laser, which evaporates the water of the hydrated matrix [ 42 - 43 ], can harm the material setting process, which continues for several hours after photoinitiated polymerization. It is impossible to wait for the material’s complete set in the clinic and then use a laser. Therefore, to simulate the clinical results in the present study, laser application was performed immediately after the samples were prepared, probably insufficient to complete the setting reaction.

Examination of failure modes showed that in the Er: YAG laser group, most failures were cohesive, and in the Er, Cr: YSGG laser, both mixed and cohesive modes were present. Despite more stains and the prevalence of cohesive failure in the Er: YAG laser group, it is possible that the Er: YAG laser might have affected the chemical structure of the material, reducing its tensile strength despite the proper porosity created by the Er: YAG laser. To review the results more accurately in similar studies on surface roughness and its effect on bond strength, direct roughness assessment methods should be used. 

## Conclusion

The results showed that the premature use of acid etching on RMGI in the clinic could harm its bond strength to the resin. Lasers are not recommended due to their high cost, low impact on increasing bond strength, and possible interference with the material's chemical structure. Using a dental bur or air abrasion can result in good outcomes.

## Conflict of Interest

The authors declare that they have no conflict of interest.
